# Exploring Telomere Association in Donor–Recipient Pairs: Implications for Kidney Graft Longevity

**DOI:** 10.3390/life16020216

**Published:** 2026-01-28

**Authors:** Zeinab Abdelrahman, Alexander P. Maxwell, Amy Jayne McKnight

**Affiliations:** Centre for Public Health, Queen’s University Belfast, Belfast BT12 6BA, UK; z.abdelrahman@qub.ac.uk (Z.A.);

**Keywords:** transplantation, telomere length, kidney transplant, graft survival, kidney

## Abstract

Introduction: Telomeres, which protect chromosome ends, are important in cell replication and are altered by ageing. In the realm of organ transplantation, telomere length has emerged as a potential biomarker for predicting both graft survival and recipient longevity. This study explores the correlation of telomere length with transplant outcomes to assess whether longer telomere length is associated with better long-term graft function and patient survival. Methods: Telomere length (TL) was analysed in 274 European renal transplant pairs (donors/recipients). Recipient DNA was collected before and after kidney transplantation, and donor DNA just prior to transplant surgery. Results: Donor TL was not significantly associated with graft survival. Donor age was a significant predictor of graft failure (1.02, 95% CI: 1.01–1.03, *p* < 0.01). Post-transplant recipient TL had a significant association with graft survival. Longer TL was associated with an up to 90% reduction in risk of graft failure (HR = 0.10, 95% CI: 0.015–0.71, *p* = 0.02). Conclusions: In this study, kidney transplant recipients with longer telomere length demonstrated significantly better long-term graft survival. If validated in additional kidney transplant cohorts, recipient telomere length could serve as a valuable biomarker for improving graft failure risk stratification and enhancing the long-term care of transplant recipients.

## 1. Introduction

Telomeres are protective nucleotide sequences that ‘cap’ the terminal ends of chromosomes. Telomeres shorten with each cell division, and when they reach a critically short length, cells enter replicative senescence or undergo apoptosis [1]. This shortening is a natural process linked to the biological process of ageing [2]. Telomere length is influenced by genetic, environmental, and lifestyle factors, serving as a biological marker of ageing and age-related health issues [3]. It is possible that telomere length, a biomarker of cellular ageing and replicative capacity, may influence post-transplant outcomes. Associations have been reported between telomere length in donor organs and/or transplant recipients and subsequent transplant outcomes in diverse types of organ transplants. Telomere length in kidney transplant recipients may predict long-term graft function [4]. Kidney allograft cells with shorter telomeres are associated with delayed graft function, acute rejection, and chronic allograft dysfunction, suggesting telomere length assessment may be a useful method for predicting kidney transplant outcomes [4,5]. Within the realm of liver transplantation, longer telomeres in donor livers have been correlated with impaired liver regeneration and lower recipient survival [6]. This indicates that telomere length may represent a risk factor for post-transplant complications. Furthermore, shorter telomere lengths in lung transplant recipients correlate with reduced chronic lung allograft dysfunction-free survival, as well as an elevated likelihood of leucopenia and CMV viraemia [7]. This emphasises the potential value of telomere length in predicting post-transplant complications. In haematopoietic cell transplantation, longer donor leukocyte telomere length correlates with improved survival in patients undergoing transplantation for severe aplastic anaemia [8]. While telomere length is a promising biomarker for predicting transplant outcomes, it is not the sole determinant. Graft survival is also significantly affected by factors such as donor type, donor and recipient age, recipient comorbidities, human leukocyte antigen (HLA) mismatch, adherence to immunosuppressive regimens and possibly genetic variants in donor organ predisposition [9]. The human leukocyte antigen (HLA) system is important in organ transplantation because the HLA proteins act as unique identification tags for cells and tissues [10,11]. A mismatch between donor and recipient HLA types can trigger the recipient’s immune system to attack and reject the donated organ. Matching donor and recipient HLA profiles can help to minimise the risk of transplant rejection and improve longer-term transplant success [12]. Modern immunosuppressive regimens can help to minimise the risks associated with HLA mismatch. This study aims to investigate the correlation between telomere length in kidney transplant recipients and donors and its impact on graft longevity.

## 2. Materials and Methods

### 2.1. Data Source (Northern Ireland Renal Transplant Cohort)

This is a retrospective cohort study with patients transplanted in 1987 to 2005 and followed up to 2012. No consent was obtained from deceased donors. Written informed consent for research participation was obtained from transplant recipients in Northern Ireland. We analysed DNA from 274 renal transplant recipients of European heritage. Recipient DNA prior to transplantation was collected at the time of wait-listing for the procedure; donor DNA was obtained from pre-procurement blood samples just prior to organ donation, and recipient DNA post-transplantation was obtained more than three months after transplantation. DNA samples were stored at 80 °C in multiple aliquots after extraction from whole blood utilising the salting out technique and normalised by PicoGreen quantitation [13,14]. All subjects identified as individuals of European heritage. Tissue-matching DNA samples were gathered before transplants between 1986 and 2005, and excess diagnostic material was stored for analysis. Informed consent was obtained for post-transplant samples (2012–2014), which were collected for longitudinal biomarker studies, based on the accessibility of those attending transplant clinics. The ethical approval reference numbers for the Belfast Renal Transplant samples are ORECNI 08/NIR03/79 and 12/NI/0178.

### 2.2. Data Collection

Baseline characteristics include donor and recipient age and sex, the recipient’s primary renal diagnosis, pre-existing diabetes mellitus (DM), new-onset diabetes after transplantation (NODAT), and immunosuppressive regimens. Transplant-related factors considered were donor–recipient sex matching, HLA compatibility, and HLA-DR locus mismatch, and graft outcome was defined as kidney graft survival, measured as the time from transplantation until graft failure (return to dialysis or re-transplantation) or patient death.

### 2.3. Telomere Length Measurement

Telomere length (TL) was assessed using monochrome quantitative polymerase chain reaction (qPCR), evaluating the ratio of telomeric repeat sequences to a single-copy reference gene (36B4) in DNA extracted from whole blood [15]. Each PCR reaction was performed in triplicate on 384-well plates, utilising 10 ng of genomic DNA, 5 μL of 2× LightCycler^®^ 480 SYBR Green I Master Mix (ScienCell Research Laboratories, CalTag Medsystems, Buckingham UK), and primers at a final concentration of 300 nM. The primers specific to telomeres were Telo1 (5′-CGGTTTGTTTGGGTTTGGGTTTGGGTTTGGGTTTGGGTT-3′) and Telo2 (5′-GGCTTGCCTTACCCTTACCCTTACCCTTACCCTTACCCT-3′). The primers employed for the 36B4 gene were as follows: 36B4d (5′-CCCATTCTATCATCAACGGGTACAA-3′) and 36B4u (5′-CAGCAAGTGGGAAGGTGTAATCC-3′). A reference DNA sample was utilised to create a standard curve, which was also assessed in triplicate. Thermal cycling was performed using the 7900 Real-Time PCR System (Applied Biosystems, Foster City, CA, USA) under the following conditions: initial hold at 50 °C for 2 min, followed by 10 min at 95 °C; 40 cycles of 15 s at 95 °C and 1 min at 60 °C; then 15 s at 95 °C, 1 min at 60 °C, 30 s at 95 °C; and a final step of 15 s at 60 °C. Data analysis was carried out using SDS V2.4.1 software (Applied Biosystems), which calculated telomere and 36B4 values for each sample based on the standard curve. Statistical analyses were performed using IBM SPSS Statistics 21 (SPSS Inc., Chicago, IL, USA) and RStudio version (2025.05.1+513).

### 2.4. Outcome Measures

The primary endpoint was kidney graft survival, defined as time from transplantation to graft failure (return to dialysis or re-transplantation) or patient death.

### 2.5. Statistical Analysis

Continuous variables were summarised as means ± standard deviation (SD), and categorical variables as frequencies and percentages. Group comparisons were performed using Student’s t-test or chi-square test, as appropriate. Pearson’s correlation coefficients were calculated to examine associations between TL and age. Donor TL was stratified into tertiles (Short, Medium, Long) for subgroup analyses. Graft survival across TL categories was evaluated using Kaplan–Meier survival analysis with log-rank testing. Multivariable Cox proportional hazard models were constructed to assess independent predictors of graft survival. Model 1 included recipient TL, while Model 2 incorporated both donor and recipient TL. Both models were adjusted for donor and recipient age, sex, HLA compatibility, DR mismatch, DM, and NODAT. Models were assessed using the concordance index (C-index), and overall model fit was evaluated with the global likelihood ratio test. Statistical significance was defined as a two-sided *p* < 0.05. Analyses were performed using RStudio version (2025.05.1+513).

## 3. Results

### 3.1. Characteristics of Study Participants

**Figure 1:**
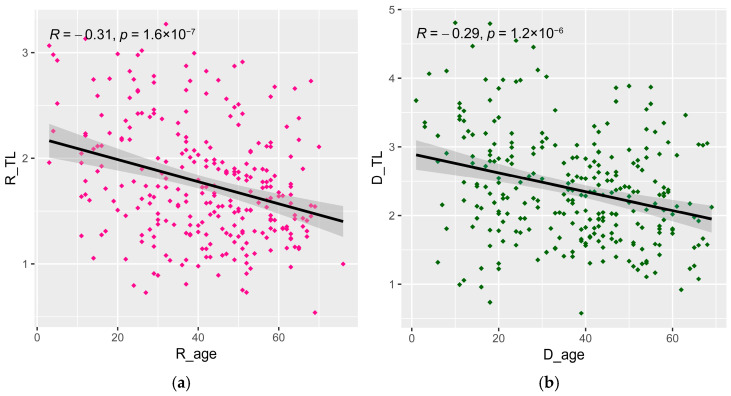
The correlation between the relative telomere length and age in (**a**) recipients and (**b**) donors. Pearson correlation and *p* values are shown. Dots represent individual sample measurements. The solid line shows the fitted linear regression, and the grey band represents the 95% confidence interval of the regression estimate.

Patients in this study were predominantly white (99.3%) with a similar sex distribution between groups (transplant recipients 61.3% male; kidney donors 59.1% male, Table 1). Recipients underwent transplantation at a mean age of 36.9 years (SD; 16.7). Donors’ mean age was 41.5 years (SD; 16.4). The relative telomere length (TL) was inversely correlated with age in both recipients (R: −0.31; *p* < 0.001, Figure 1a) and donors (R: −0.29; *p* < 0.001, Figure 1b). Even though donors were older, kidney transplant recipients had significantly shorter telomere lengths (mean [SD] 1.76 [0.56]), than donors (2.39 [0.79]), even after adjusting for age (adjusted mean telomere length difference comparing recipients with donors is −0.57; *p* < 0.001).

**Figure 2:**
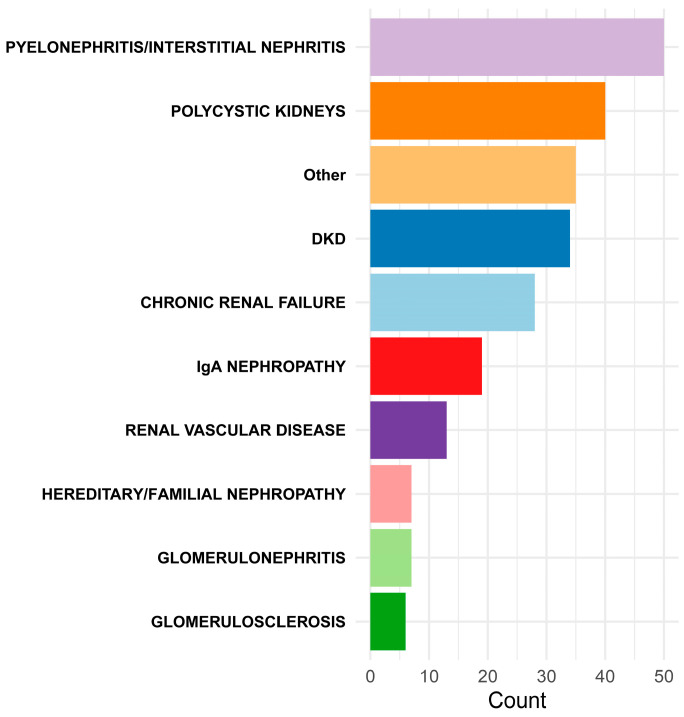
Top diagnoses among kidney transplant recipients included in the study. Diagnoses are ranked by frequency, with pyelonephritis/interstitial nephritis being the most common. Other diagnoses include various forms of chronic renal failure, polycystic kidney disease, IgA nephropathy, diabetic kidney disease (DKD), renal vascular disease, nephropathy, glomerulonephritis, glomerulosclerosis, and other conditions with fewer than three cases, which are not reported for disclosure reasons. Diagnostic classifications are based on clinical records and, where applicable, histopathological confirmation.

The most common primary diagnoses were pyelonephritis/interstitial nephritis (50 cases, 18.2%) and polycystic kidneys (40 cases; 14.6%) and other diagnoses included diabetic kidney disease (34 cases; 12.4%), chronic renal failure of uncertain aetiology (28 cases; 10.2%), IgA nephropathy (19 cases; 6.9%), and renal vascular disease (9 cases; 3.3%), Figure 2.

Table 2 presents a comparative analysis of kidney transplant recipients categorised into tertiles based on donor relative telomere length. The three groups are defined as follows: first tertile (short, TL ratio (T/S) < 1.97, *n* = 189), second tertile (medium, T/S 1.97–2.72, *n* = 63), and third tertile (long, T/S ≥ 2.72, *n* = 21). The relative telomere length increased significantly across tertiles (short: 1.47 ± 0.30; medium: 2.29 ± 0.21; long: 2.90 ± 0.15; *p* < 2 × 10^−16^). Transplant recipient age showed a significant decreasing trend with increasing donor TL (short: 44.5 ± 15.0 years; medium: 36.6 ± 17.0 years; third: 28.8 ± 17.0 years; *p* < 0.001). Sex matching between recipients and donors, as well as the proportion of male recipients, did not differ significantly across tertiles (*p* = 0.79 and *p* = 0.3, respectively). Favourable HLA matching and DR locus matching were comparable across tertiles, with the highest frequency of matches at the first tertile (*p* = 0.80 and *p* = 0.52, respectively). HLA mismatching distribution (0–5 mismatches) showed no significant variation (*p* = 0.73). Functioning grafts with active follow-up were reported in 90 (first tertile), 29 (second tertile), and 9 (third tertile) recipients. Graft failure with subsequent patient death on dialysis occurred in 27 (first tertile), 7 (second tertile), and 1 (third tertile) case. Graft failure in patients alive on dialysis was observed in 26 (first tertile), 17 (second tertile), and 6 (third tertile) recipients. Graft failure was reported in only one patient in the second tertile, and the patient was alive upon transfer outside the renal unit. The number of patients who died with a functioning graft was 46 (first tertile), 9 (second tertile), and 5 (third tertile).

CNI-based immunosuppression regimens were used in 140 recipients in the first tertile, 49 recipients in the second tertile, and 14 recipients in the third tertile. Among kidney transplant recipients, diabetes mellitus (DM) and new-onset diabetes after transplantation (NODAT) were evaluated across tertiles of donor TL. The number of pre-existing DM cases was highest in the first tertile (n = 26), with fewer cases in the second (n = 5) and third (n = 3) tertiles. The number of recipients without any form of diabetes (neither DM nor NODAT) was 154, 53, and 16 in the first, second, and third tertiles, respectively. NODAT alone was observed in 9, 5, and 2 recipients across the tertiles. The mean follow-up time to 2012 was 206 ± 57.7, 200 ± 62.3, and 214 ± 73.1 months, respectively (*p* = 0.61 for both time points).

### 3.2. Recipient Pre-Transplant Telomere Length and Post-Transplant Outcome

**Figure 3:**
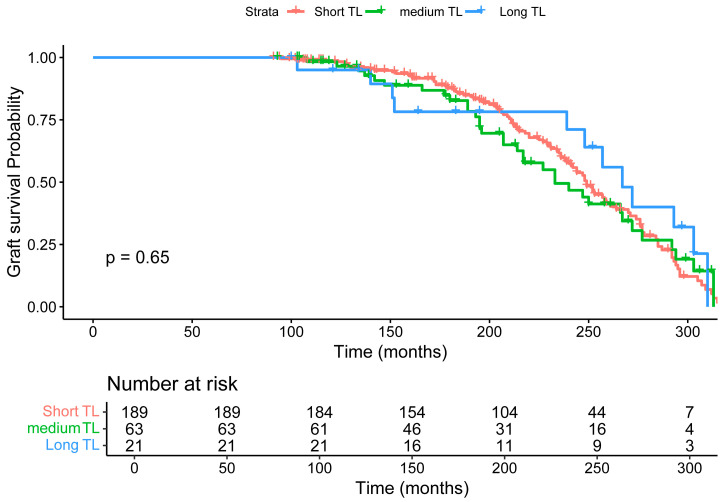
Graft survival probability for kidney transplant recipients, categorised by relative donor telomere length: Short TL (red, n = 189, T/S < 1.97), Medium TL (green, n = 63, T/S 1.97–2.72), and Long TL (blue, n = 21, T/S ≥ 2.72). Graft survival probabilities were estimated over a follow-up period of up to 300 months (25 years). Censoring events are indicated by plus signs. The number of patients at risk at each time point is shown below the *x*-axis.

We first evaluated the association between pre-transplant donor telomere length (TL) and graft survival using Kaplan–Meier analysis. Recipients were stratified into tertiles based on donor TL: Short TL (n = 189, T/S < 1.97), Medium TL (n = 63, T/S 1.97–2.72), and Long TL (n = 21, T/S ≥ 2.72). Over a follow-up period of up to 300 months (~25 years), all three groups exhibited a gradual decline in graft survival. The log-rank test yielded a *p*-value of 0.65, indicating no statistically significant difference in graft survival among the groups (Figure 3).

**Figure 4:**
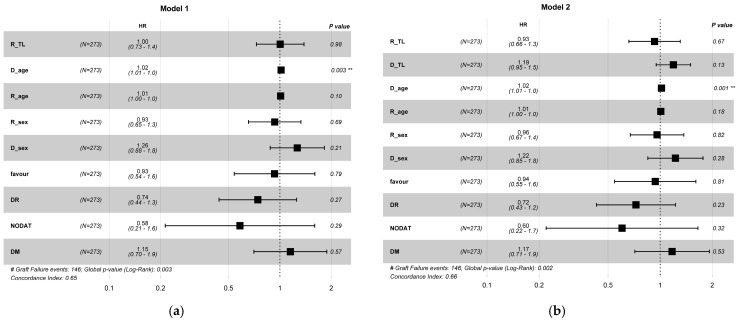
Multivariable Cox proportional hazard model forest plots for graft survival. Model (**a**) does not include donor telomere length (D_TL), while Model (**b**) includes donor telomere length along with recipient telomere length (R_TL). Both models adjust for donor and recipient age and sex, HLA matching (favourable vs. unfavourable), DR mismatch, new-onset diabetes after transplantation (NODAT), and pre-existing diabetes mellitus (DM). The forest plots display hazard ratios (HRs) and 95% confidence intervals (CIs) for each variable. A black dashed line at HR = 1 indicates no effect. ** *p* < 0.01.

Next, we assessed pre-transplant recipient TL using multivariable Cox proportional hazards models. Donor telomere length was used as a covariate in the second model, but not in the first one. Both models were adjusted for key clinical and demographic variables, including recipient age, sex, diabetes status (DM), new-onset diabetes after transplant (NODAT), donor age and sex, HLA matching, and immunological risk (DR mismatch). Within Model 1, the association between recipient telomere length and graft survival was not statistically significant (HR = 1.00, 95% CI: 0.73–1.38). Donor age was significantly correlated with elevated risk of graft failure (HR = 1.02, 95% CI: 1.01–1.03, *p* = 0.003). Statistical significance was observed in the overall model (*p*-value = 0.003), accompanied by a concordance index of 0.65 (Figure 4a). In Model 2, recipient telomere length did not achieve statistical significance (HR = 0.93, 95% CI: 0.66–1.31). Donor telomere length did not show a significant association with graft survival (HR = 1.19, 95% CI: 0.95–1.50). Donor age was still a significant predictor (HR = 1.02, 95% CI: 1.01–1.03, *p* =0.001). The model demonstrated comparable performance (concordance index = 0.66) and exhibited statistical significance (*p* = 0.002, Figure 4b).

### 3.3. Recipient Post-Transplant Telomere Length and Post-Transplant Outcome

**Figure 5:**
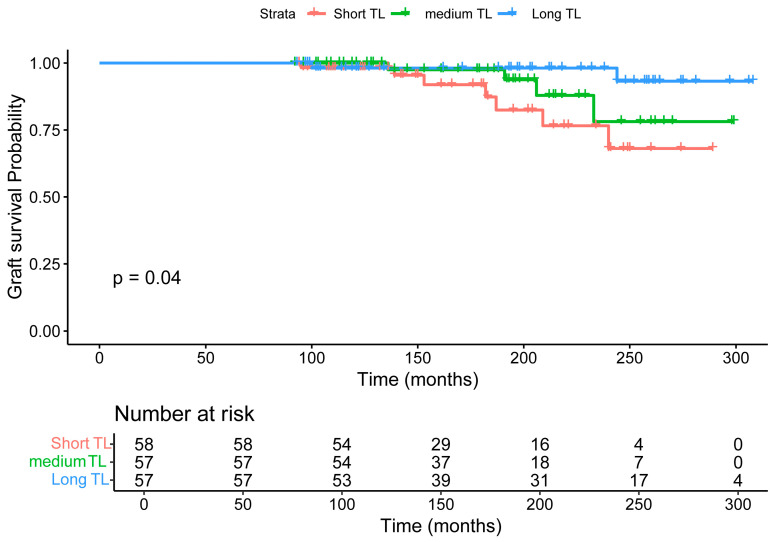
Kaplan–Meier survival curves stratified by post-transplant relative telomere length (TL). Patients were grouped into tertiles based on post-transplant TL: Short TL (red, n = 58, T/S < 0.62), Medium TL (green, n = 57, T/S 0.62–0.94), and Long TL (blue, n = 57, T/S > 0.94). Graft survival probabilities were estimated over a follow-up period of up to 300 months (25 years). The Long TL group demonstrated significantly better long-term graft survival compared to the Medium and Short TL groups (log-rank *p* = 0.04). Censoring events are indicated by plus signs. The number of patients at risk at each time point is shown below the *x*-axis.

Given that neither donor TL nor recipient pre-transplant TL showed a significant association with graft survival (Figure 3 and Figure 4), we next investigated whether post-transplant TL was associated with graft survival. As shown in Figure 5, recipients were stratified into three tertiles: Short TL (n = 58, T/S < 0.62), Medium TL (n = 57, T/S 0.62–0.94), and Long TL (n = 57, T/S > 0.94). Over the course of follow-up (up to 300 months, approximately 25 years), the Long TL group exhibited significantly improved graft survival compared to the Medium and Short TL groups. The survival curves diverged around 100 months, with the Short TL group showing the steepest decline in graft survival probability, and the Long TL group maintaining the highest survival rates throughout long-term follow-up. The difference in graft survival between the groups was statistically significant (log-rank *p* = 0.04, Figure 5).

**Figure 6:**
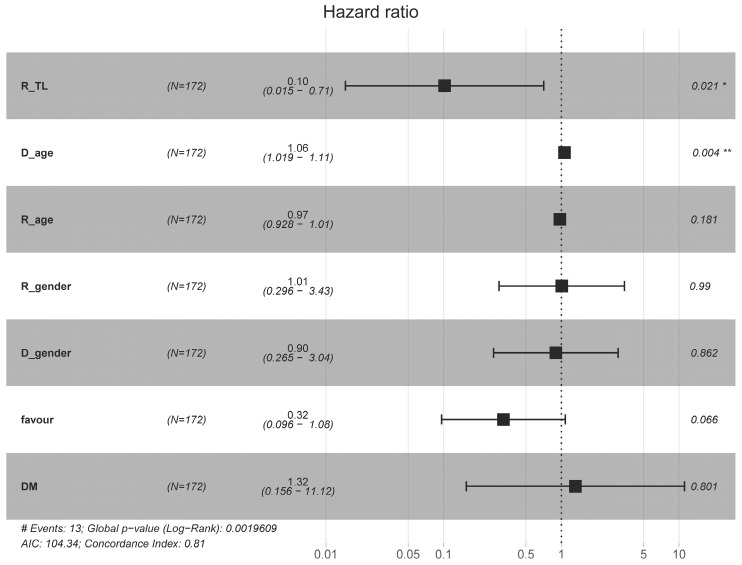
Forest plot displaying the hazard ratios (HRs) for graft survival based on multivariable Cox proportional hazard analysis. Variables included in the model were recipient and donor age, gender, post-transplant recipient relative telomere length (R_TL), immunologic risk status (favourable) and pre-existing diabetes mellitus (DM). Each row presents the HR and its 95% confidence interval (CI) for the corresponding variable. Relative telomere length was significantly associated with improved graft survival (HR = 0.10; 95% CI: 0.01–0.71; *p* = 0.021), indicating a 90% reduced risk of graft failure for patients with longer telomeres. Donor age was also significantly associated with outcome (HR = 1.06; 95% CI: 1.02–1.11; *p* = 0.004), suggesting increased risk in older donor age. The forest plots display hazard ratios (HRs) and 95% confidence intervals (CIs) for each variable. A black dashed line at HR = 1 indicates no effect. * *p* < 0.05, ** *p* < 0.01.

Consistent with the Kaplan–Meier analysis, which demonstrated significantly improved graft survival among recipients with longer post-transplant TL, we further evaluated this association using a multivariable Cox proportional hazard model. In this model, post-transplant recipients’ relative telomere length (R_TL) and donor age (D_age) emerged as significant factors. Patients with longer post-transplant TL (T/S > 0.94) had significantly improved graft survival (HR = 0.10, 95% CI: 0.015–0.71, *p* = 0.021), indicating a 90% reduction in the hazard of graft failure compared to those with shorter telomeres. Donor age was also significantly associated with increased risk of graft failure, with each additional year of donor age increasing the hazard by approximately 6.1% (HR = 1.062, 95% CI: 1.02–1.12, *p* = 0.004). Other covariates, including recipient age, recipient and donor gender, and favourable HLA match, were not statistically significant (*p* > 0.05). The model demonstrated good discriminative ability with a concordance index (C-index) of 0.81, and the global likelihood ratio test was significant (*p* = 0.002), indicating the model explains a meaningful portion of the variability in graft outcomes (Figure 6).

## 4. Discussion

In kidney transplantation, the relationship between donor telomere length and recipient characteristics highlights a complex interplay between biological ageing and disease risk. The results indicate that kidney transplant recipients exhibited significantly shorter telomere lengths than their donors, which is consistent with previous research associating chronic and end-stage renal disease with accelerated telomere shortening [16,17]. Recipients with longer TL were significantly younger and had a lower prevalence of pre-existing diabetes. This supports the notion that TL serves as a marker for biological age, as longer telomeres are usually present in younger individuals and associated with a lower incidence of age-related illnesses, including diabetes [18]. Additionally, these findings support the hypothesis that biological age, as indicated by TL, contributes to transplant outcomes [19].

Importantly, donor TL did not significantly influence graft survival in either unadjusted or adjusted models. Survival analysis showed no significant difference in graft survival across donor TL tertiles, and Cox regression confirmed that donor TL was not an independent predictor. The lack of a significant association between donor telomere length and graft survival should be interpreted with caution. The donor TL analysis was limited by the small recipient sample size in the Long TL tertile (n = 21), which may have reduced statistical power to detect subtle effects. Future studies with larger, carefully phenotyped cohorts and standardised TL assessments are needed to clarify the role of donor TL in graft outcomes. Donor age, however, consistently emerged as a significant risk factor for graft failure, aligning with previous studies demonstrating poorer outcomes with older donor kidneys [20,21].

Recipient TL, particularly post-transplant, was significantly associated with graft survival. Recipients with longer TL post-transplant had markedly improved outcomes, with a 90% reduction in the hazard of graft failure. This supports the hypothesis that TL reflects biological resilience and regenerative capacity, which may be critical for long-term graft function [22]. The finding that recipient TL was more strongly associated with graft survival than donor TL justifies the investigation of potential underlying mechanisms. It is conceivable that recipient TL reflects systemic biological ageing and overall tissue repair capacity, which may impact recovery and long-term graft function. A longer TL may be associated with an increased resistance to immunological stress and improved response to immunosuppressive therapy, while a shorter TL may indicate reduced regenerative capacity and increased susceptibility to complications such as chronic rejection or infection [23]. Furthermore, post-transplant TL could reflect the cumulative impact of immunosuppression, inflammation, and metabolic stress following transplantation, serving as a dynamic marker of post-operative physiological status rather than a static pre-transplant feature. Future research incorporating longitudinal measurements of TL and immune competence markers may elucidate these mechanisms.

Limitations include relatively small sample sizes in the Medium and Long donor TL tertiles, which may reduce statistical power in the absence of a replication cohort. We did not find a similar cohort with telomere length already measured in recipients pre- and post-transplant as well as donors in which to replicate our findings. Additionally, the retrospective nature of the cohort and the historical follow-up period (ending in 2012) do not capture more recent outcomes; however, the population has a sufficient mean of 205.1 months and a maximum of 317 months follow-up period.

Immunosuppressive regimens and clinical practices (including donor selection criteria) have changed considerably since the period 1987–2012 when DNA samples were obtained and follow-up completed for our retrospective study. Tacrolimus has replaced cyclosporine as the dominant calcineurin inhibitor (CNI) in light of its better efficacy. Similarly, mycophenolate mofetil has become the preferred antiproliferative agent as it proved more effective than azathioprine. Over the last 15 years, there has been much wider use of antibody-based induction therapy and a greater focus on minimisation strategies to limit CNI nephrotoxicity and steroid side effects. This evolution of immunosuppressive regimens means that our findings of improved survival of kidney transplant with longer telomere length cannot be directly extrapolated to the modern era of clinical practice.

Finally, although more than 95% of the kidney transplant recipients remained in Northern Ireland for the duration of the study period, post-transplant TL measurements were only available for recipients who attended follow-up clinics, which may introduce some selection bias and limit generalizability. This highlights the importance of routine, regular post-transplant follow-up for all recipients to ensure optimal care and data collection. Therefore, future research should validate these findings in larger, multi-centre cohorts that reflect current clinical practice and explore whether post-transplant TL can be established as a clinically actionable prognostic biomarker in kidney transplantation.

## 5. Conclusions

While donor TL does not independently predict graft survival, post-transplant recipient TL may serve as a valuable biomarker of long-term transplant success. Recipients with longer TL have better survival, even after adjusting for recipient age. To establish clinical utility, future research should include large, multi-centre prospective studies to guide risk stratification and assess whether TL interacts with established risk factors such as donor age and immunological mismatch. Additionally, exploring longitudinal changes in TL may reveal whether dynamic measurements provide greater prognostic insight than a single time point.

## Figures and Tables

**Table 1:** Characteristics of kidney transplant recipients and donors.

	Recipients (n = 274)	Donors (n = 274)	*p* Value
Age (mean ± SD)	36.9 ± 16.7	41.5 ± 16.4	*p* < 0.001
Age in decades			
0–19	31	53	
20–29	40	45	
30–39	45	41	0.03
40–49	58	60	
50–59	61	52	
60+	39	22	
Sex (men)	168 (61.3%)	162 (59.1%)	0.12
Relative telomere length (mean ± SD)	1.76 ± 0.56	2.39 ± 0.79	*p* < 0.001

**Table 2:** Characteristics of kidney transplant recipients by donor relative telomere length categories.

	T/S < 1.97 (n = 189)	T/S 1.97–2.72 (n = 63)	T/S ≥ 2.72 (n = 21)	*p* Value
Relative telomere length (mean ± SD)	1.47 ± 0.30	2.29 ± 0.21	2.9 ± 0.15	*p* < 0.001
Age (mean ± SD)	44.5 ± 15.0	36.6 ± 17.0	28.8 ± 17.0	*p* < 0.001
recipients/donors sex match				
Male/Male	75	24	7	
Male/Female	46	13	3	0.79
Female/Male	34	15	6	
Female/Female	34	11	5	
Sex (men)	121	37	10	0.30
Favourable HLA match	111	34	12	0.80
Match at DR locus	102	39	11	0.52
Number of HLA mismatching				
0	19	4	2	
1	20	12	3	
2	71	19	8	0.73
3	64	22	8	
4	13	6	0	
5	2	0	0	
Functioning graft, alive (active follow-up)	90	29	9	
Graft failure and the patient died on dialysis	27	7	1	
Graft failure, patient alive on dialysis	26	17	6	0.14
Graft failure, patient alive and transferred	0	1	0	
Patient died with a functional graft	46	9	5	
Immunosuppressants (CNI, yes)	140	49	14	0.75
No DM or NODAT	154	53	16	
NODAT only	9	5	2	0.15
Pre-transplant DM	26	5	3	
Follow-up time to 2012 (months, mean ± SD)	206 ± 57.7	200 ± 62.3	214 ± 73.1	0.62
Follow-up time to 2012 of survivors (months, mean ± SD)	183 ± 53.7	184 ± 68.3	194 ± 74.7	0.86

## Data Availability

The data supporting the conclusions of this article will be made available by the authors on request.
